# Mechanisms of carcinogenic activity triggered by lysine-specific demethylase 1A

**DOI:** 10.3389/fphar.2022.955218

**Published:** 2022-07-19

**Authors:** Chao Yang, Dan Li, Shaohong Zang, Lei Zhang, Zhangfeng Zhong, Yingtang Zhou

**Affiliations:** ^1^ National Engineering Research Center for Marine Aquaculture, Institute of Innovation and Application, Zhejiang Ocean University, Zhoushan, China; ^2^ State Key Laboratory of Southwestern Chinese Medicine Resource, School of Pharmacy, Chengdu University of Traditional Chinese Medicine, Chengdu, China; ^3^ Department of Chemical Engineering, Waterloo Institute for Nanotechnology, University of Waterloo, Waterloo, ON, Canada; ^4^ Macau Centre for Research and Development in Chinese Medicine, Institute of Chinese Medical Sciences, University of Macau, Taipa, Macao SAR, China

**Keywords:** anticancer activity, demethylation, epigenetics, histone modifications, LSD1, lysine methylation, signaling pathway, targets

## Abstract

Epigenetics has emerged as a prime focus area in the field of cancer research. Lysine-specific demethylase 1A (LSD1), the first discovered histone demethylase, is mainly responsible for catalysing demethylation of histone 3 lysine 4 (H3K4) and H3K9 to activate or inhibit gene transcription. LSD1 is abnormally expressed in various cancers and participates in cancer proliferation, apoptosis, metastasis, invasion, drug resistance and other processes by interacting with regulatory factors. Therefore, it may serve as a potential therapeutic target for cancer. This review summarises the major oncogenic mechanisms mediated by LSD1 and provides a reference for developing novel and efficient anticancer strategies targeting LSD1.

## 1 Introduction

Cancer is a life-threatening disease that seriously threatens human health and life (Liu et al., 2020). The number of new cancer cases exceeded 19.29 million in 2021, with approximately 9.96 million deaths reported worldwide (Siegel et al., 2021). Mechanisms underlying tumorigenesis are usually driven by genetic mutations, especially mutations that occur in cancer suppressor genes and (or) proto-oncogenes (Wang et al., 2018; Fang et al., 2020). Several recent studies have shown that abnormal epigenetic modifications, such as DNA and histone modifications, play essential roles in regulating tumorigenesis and the proliferation and differentiation of cancer stem cells (Chen J. et al., 2020; Ghasemi et al., 2021; Zhao and Peng, 2022). In addition, epigenetic reprogramming and unlocking phenotypic plasticity have been reported as the hallmarks of cancer (Gupta et al., 2019; Hanahan, 2022; Saltarella et al., 2022).

**FIGURE 1:**
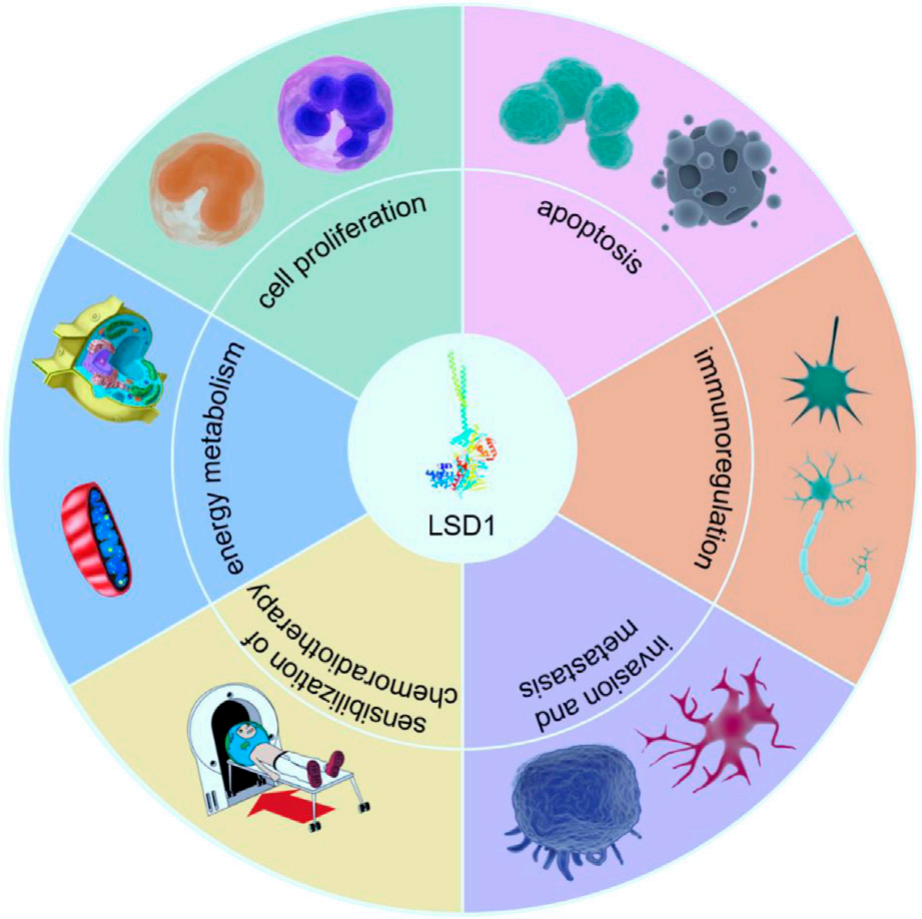
The role of LSD1 in tumorigenesis and development. LSD1 participates in cancer progression by regulating multiple critical physiological processes, such as the proliferation, invasion, metastasis, energy metabolism, immune regulation, and drug resistance of cells (drawn using tools from PNGBAG; https://www.pngbag.com/, copyright ^©^2020 PNGBAG.COM).

Histone octamers are composed of two H2A, H2B, H3, and H4 subunits, and each core histone has a folding region and an amino-terminal domain. Various covalent modifications such as acetylation, phosphorylation, methylation, ubiquitination, and glycosylation can occur at the amino terminus of histones, which affect the chromatin structure and activation or inhibition of transcription (Yang et al., 2021a; Yang et al., 2021b). Shi and Tsukada (2013) discovered the first histone demethylase in 2004, a lysine-specific histone demethylase 1 (LSD1). The discovery of LSD1 indicates that histone methylation modification is a dynamic process that can regulate not only the methylation of histones but also the interaction of histones with other functional proteins. Numerous studies have shown that LSD1 is responsible for regulating the transcriptional activation and repression of specific genes, X chromosome inactivation and viral pathogenesis and plays an essential role in embryonic and cancer development (Miller et al., 2021). This review summarises the research progress of LSD1 in various cancers and highlights the mechanism of action of LSD1 underlying its anticancer activity (Figure 1).

## 2 Structure and biological function of LSD1

**FIGURE 2:**
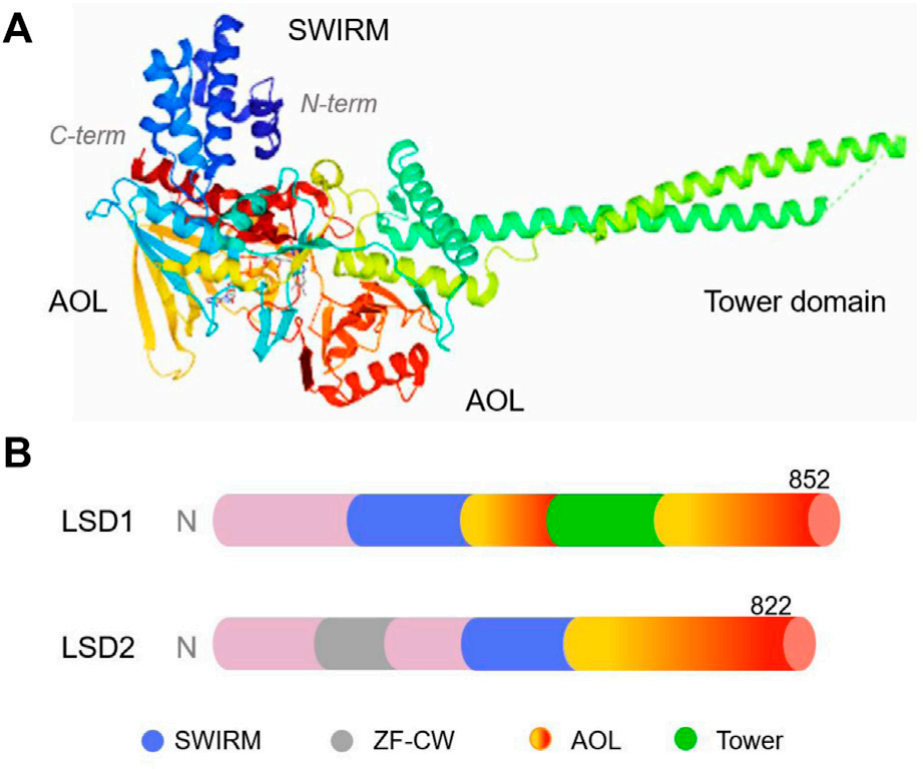
The structure of human lysine-specific demethylase (LSD) protein. **(A)** Three-dimensional structure of human LSD1 protein (PDB No.: 2Z5U), Copyright ^©^ 2007 Elsevier Inc. **(B)** Schematic illustration of the structure of LSD1 and LSD2.

LSD1, also known as KDM1A, AOF2, BHC110, KIAA0601, NPAO, and p110b, is a flavin adenine dinucleotide (FAD)-dependent monoamine oxidase located in the nucleus (Anand and Marmorstein, 2007). It mainly consists of three domains, namely, the N-terminal SWIRM (Swi3p/Rsc8p/Moira), C-terminal amine oxidase (AOL) and central Tower domains (Figure 2) (Karakaidos et al., 2019; Majello et al., 2019). The SWIRM domain is highly conserved and can recognise and bind to histones, whereas the AOL domain mainly binds to histone substrates (Chen et al., 2006; Castelli et al., 2018). The Tower domain extends two parallel alpha helices outward from the AOL domain to facilitate interactions with other co-regulators/cofactors, including FAD. Typically, the SWIRM and AOL domains are connected and overlap to form a spherical shape (Chen et al., 2006; Kaniskan et al., 2018). The Tower domain is formed by two helices extending outward from the C-terminal AOL domain, thereby dividing the AOL domain into the following two parts: the FAD-binding domain and a catalytically active centre. The main difference between LSD1 and LSD2 is that the N-terminus of LSD2 has a zinc finger domain (Zn-CW), which is necessary for binding to its methylated substrate (Zhang et al., 2013). LSD1+8a, a subtype generated by alternative splicing of LSD1, is involved in the differentiation of neuronal cells *via* demethylation of H3K9me2/1 (Jotatsu et al., 2017).

The primary biological function of LSD1 is the demethylation of histone or non-histone proteins (Gu et al., 2020). Some standard arginine and lysine methylation sites on histones or non-histones proteins include histone 3 lysine 4 (H3K4), H3K9, H3K27, H3K36, H3K79, and histone 4 lysine 20 (H4K20). Methylation at H3K4, H3K36, and H3K79 sites is involved in the repression of gene transcription (Hyun et al., 2017). LSD1 can remove the monomethyl (Me1) or dimethyl (Me2) group from lysine residues and can specifically demethylate the H3K4 site to inhibit its transcriptional activity (Wang and Cole, 2020). In addition, LSD1 can interact with REST/CoREST/co-regulatory factors to exert a regulatory effect (non-methylation function) (Gu et al., 2020; Perillo et al., 2020).

## 3 Primary targets or signalling pathways mediated by LSD1

**FIGURE 3:**
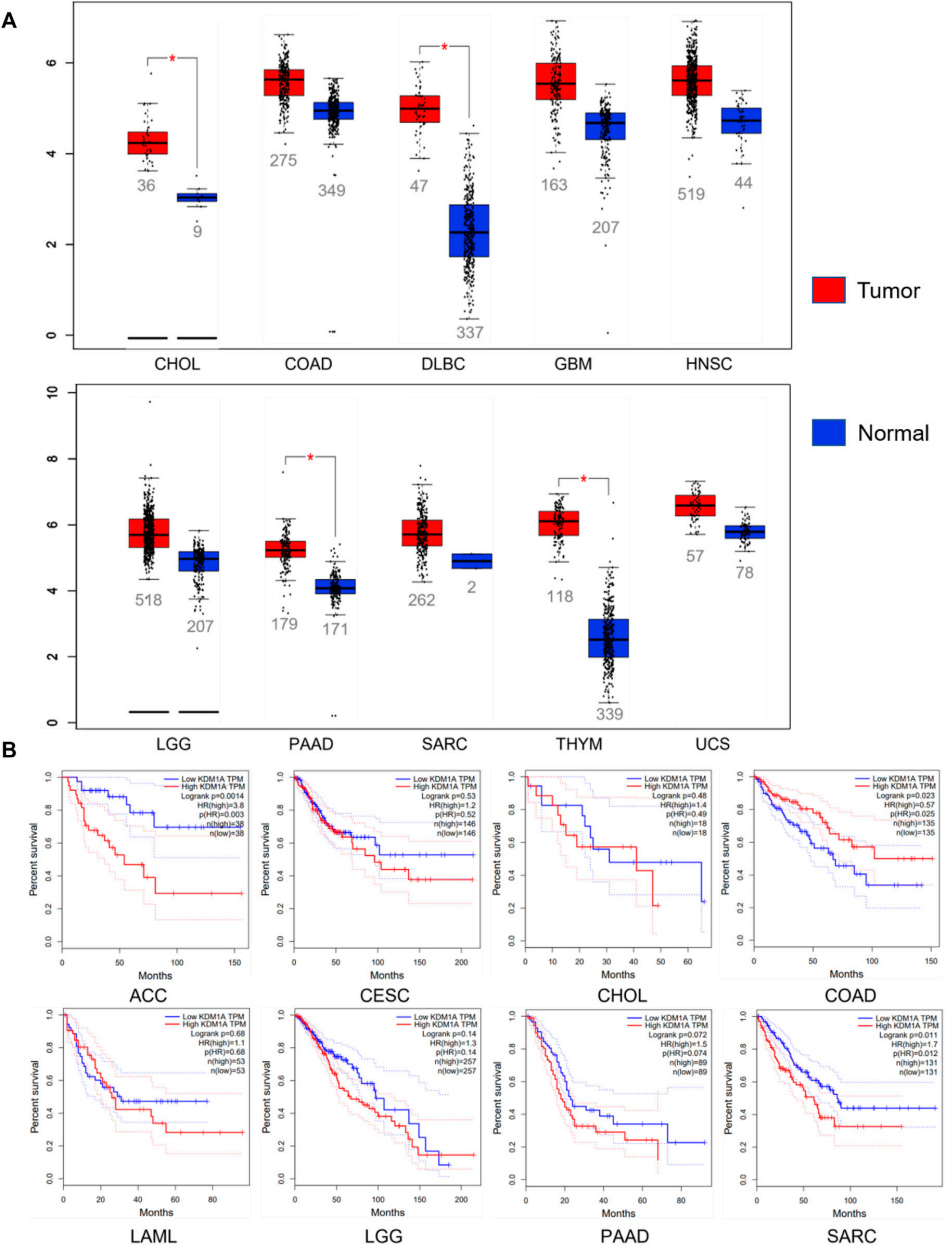
Relationship between LSD1 and cancers. **(A)** LSD1 is aberrantly expressed in various cancers. **(B)** Analysis of the correlation between LSD1 expression and overall survival (OS). Data from the GEPIA database, http://gepia.cancer-pku.cn/index.html.

Abnormal expression of LSD1 in various cancers promotes cancer progression and is closely related to the survival and prognosis of patients (Figure 3) (Ramírez-Ramírez et al., 2020; Zhang et al., 2020). Given that LSD1 is a potential therapeutic target for cancer, many targeted inhibitors of LSD1 with excellent anticancer activity have been reported, and some of these inhibitors have entered clinical trials (Maes et al., 2018; Wimalasena et al., 2020; Fang et al., 2021; Yang et al., 2022).

### 3.1 LSD1-mediated tumor regulators

#### 3.1.1 p53

p53 is a well-known cancer suppressor and transcriptional activator regulated by many post-translational modifications, including lysine methylation. LSD1 promotes the interaction of p53 with the coactivator 53BP1 (p53-binding protein 1) by removing the monomethyl (K370me1) and dimethyl (K370me2) groups at K370 (Chen L. et al., 2020). However, the direct interaction of LSD1 with p53 inhibits p53-mediated transcriptional activation and apoptosis, resulting in altering the chromatin structure and inhibiting the development of the cancer marker alpha-fetoprotein (AFP) (Wen and Wang, 2022). Therefore, downregulating the expression of LSD1 and (or) inhibiting its activity can promote cancer cell apoptosis. The intrinsically disordered C-terminal domain of p53 inhibits LSD1 activity, and direct interaction between the two proteins may contribute to their functional crosstalk (Speranzini et al., 2017).

Overexpression of LSD1 can strongly inhibit p53 in prostate cancer (PCa) and promote androgen-independent (AI) transformation of PCa and LNCaP cells in an androgen-deficient setting (Li X. et al., 2016). Low doses of the LSD1 inhibitor HCI-2509 can significantly alter the cell cycle and expression of p53, MYCN and hypoxia pathway-related genes in neuroblastoma (Gupta et al., 2018).

#### 3.1.2 Terminal deoxynucleotidyl transferase-interacting factor 1

Terminal deoxynucleotidyl transferase (TdT)-interacting factor 1 (TdIF1) is a ubiquitously expressed protein that binds to TdT polymerase (Zhang et al., 2018). It is abundantly expressed in lung cancer, is associated with a poor prognosis and is a potential cancer-promoting factor and therapeutic target (Liu et al., 2021). Silencing or inhibiting TdIF1 can inhibit the migration and invasion of cancer cells and cancer growth. LSD1 binds to TdIF1 and is recruited to the E-cadherin promoter region, where it activates transcription to induce epithelial–mesenchymal transdifferentiation (EMT) and promote the invasion and migration of cancer cells (Zhang et al., 2019; Liu et al., 2021). Therefore, simultaneous inhibition or silencing of LSD1 and TdIF1 exerts synergistic effects on anticancer proliferation, migration and invasion.

#### 3.1.3 DNA-binding zinc finger transcription factor

Growth factor independence 1 (GFI1) and the closely related protein GFI1B are major regulators of both early haematopoiesis and haematopoietic stem cells, and their aberrant activation has been implicated in human medulloblastoma and haematological malignancies (Beauchemin and Möröy, 2020; Ravasio et al., 2020). GFI1 and GFI1B are major proteins that interact with LSD1, recruit histone-modifying enzymes to the promoters and enhancers of target genes through the N-terminal SNAG domain to regulate the expression of target genes, such as HDAC and LSD1, and play an essential role in cell proliferation and differentiation (Maiques-Diaz et al., 2018; Beauchemin and Möröy, 2020; Tatsumi et al., 2020). In addition, they are involved in the recruitment of CoREST complexes to chromatin in myeloid cells (Van Bergen and Van Der Reijden, 2019). An irreversible inhibitor of LSD1, T-3775440, selectively suppressed the proliferation of SCLC cells overexpressing GFI1B (Takagi et al., 2017).

#### 3.1.4 Polo-like kinase 1

Polo-like kinase 1 (PLK1) is a serine/threonine kinase that is a key regulator of eukaryotic cell division. Because PLK1 is highly expressed in cancer cells and is associated with the poor prognosis of many cancers, it has emerged as a new target for developing many anticancer drugs (Li Z. et al., 2020; Yu et al., 2021).

LSD1 can recognise and bind to the promoter region of PLK1 to regulate its expression, which regulates the expression of cell division-related genes. However, the interaction between p53 and PLK1 is negatively regulated (Li Y. et al., 2020; Jung et al., 2021). p53 inhibits transcription at the PLK1 promoter, whereas PLK1 inhibits the function of p53 by directly binding to it or inactivating it by promoting its degradation (Dufies et al., 2021; Zhang C. et al., 2022; Hirschler-Laszkiewicz et al., 2022). Therefore, LSD1 may regulate the cell cycle and proliferation through the p53/PLK1 signalling axis.

#### 3.1.5 Hypoxia-inducible factor-1α

Hypoxia-inducible factor 1-alpha (HIF-1α) is a key protein that regulates the expression and synthesis of cytokines and growth mediators in cells during hypoxia (Yang et al., 2021c). It plays an essential role in the formation of tumor blood vessels and the proliferation, metastasis, invasion, apoptosis, energy metabolism, and drug resistance of tumor cells (Li G. et al., 2021). The expression of HIF-1α is positively correlated with the malignancy and poor prognosis of cancers. Therefore, inhibiting the expression of HIF-1α and blocking hypoxia signal transduction mediated by it may help develop novel therapeutic strategies for cancers.

The stability of HIF-1α is regulated by lysine methylation. SET7/9 methyltransferase adds a methyl group to HIF-1α, which subsequently triggers protein degradation *via* the ubiquitin–proteasome pathway. However, LSD1 demethylates HIF1α at K391, thus protecting HIF-1α from ubiquitin-mediated protein degradation, and directly inhibits PHD2-mediated hydroxylation of HIF-1α (Baek and Kim, 2016; Lee et al., 2017; Yang et al., 2017). In addition, LSD1 is highly expressed in cancers, potently stabilises HIF1α and enhances the transcriptional activity of downstream target genes, such as VEGF, which can induce cancer angiogenesis. Cellular senescence is a state of permanent cell cycle arrest, which strongly affects the development, invasion and prognosis of cancers. In glioblastoma, silencing/inhibiting LSD1 downregulates HIF-1α, which inhibits the growth and migration of cancer cells and induces cellular senescence (Saccà et al., 2019).

#### 3.1.6 Snail

Snail is an important regulator of EMT-associated zinc finger structures. It is involved in the immune escape, immune regulation, drug resistance of cancer cells, and maintenance of cancer cell stemness. And it also recruits LSD1 to suppress the expression of breast cancer susceptibility gene 1 (BRCA1) (Li H. M. et al., 2020; Pan et al., 2021). The interaction of LSD1 with the SNAG domain at the N-terminus of Snail inhibits the expression of E-cadherin.

An inhibitor of LSD1, parnate, blocks Snail-dependent inhibition of the E-cadherin promoter and inhibits the migration and invasion of cancer cells without affecting their proliferation (Ferrari-Amorotti et al., 2013; Ferrari-Amorotti et al., 2014). It has been reported that parnate treatment inhibited bone marrow homing/transplantation of Snail2-expressing K562 cells. Furthermore, the knockdown of LSD1 in triple-negative breast cancer (TNBC) can significantly inhibit the proliferation and invasion of cancer cells and metastasis (Bai et al., 2017). LSD1 interacts with Snail1 to mediate the ectopic expression of Snail1, thus increasing the risk of AML in mice (Carmichael et al., 2020).

#### 3.1.7 Zinc finger proteins

Zinc finger proteins (ZNFs) are the largest family of transcription factors in the human genome with extensive and important molecular biological functions. In addition to regulating the transcription of downstream target genes by interacting with different functional domains and trans-regulatory elements, ZNFs can recruit other chromosome modifiers and interact with different partner proteins to inhibit or promote gene transcription. ZFPs are abnormally expressed in cancer cells and participate in the occurrence and development of cancers by regulating gene transcription and translation.

ZNF217, a C2H2 zinc finger transcription factor, acts as a key effector in stimulating embryonic immortalisation and oncogenicity in various cancer-related processes. Downregulation of ZNF217 can inhibit the proliferation, invasion and EMT of HCC cells (Si et al., 2019). Mechanistic studies have shown that ZNF217 interacts with CoREST, LSD1, HDAC, and C-terminal binding protein (CtBP) to inhibit the transcriptional activity of CDH1 (Li Y. et al., 2021). SP-2509 inhibits the viability of cancer cells by blocking the binding of LSD1 to ZNF217 by inhibiting LSD1-independent function in castration-resistant PCa (Sehrawat et al., 2018).

#### 3.1.8 Immune checkpoint

Immune checkpoints are inhibitory signals in the immune system, which mainly maintain normal immune function by regulating immune activation (Sun et al., 2020; Zhang and Sun, 2020). The binding of programmed death-ligand 1/2 (PD-L1/PD-L2) expressed on cancer cells with programmed cell death protein 1 (PD-1) expressed on cytotoxic T cells triggers inhibitory signalling, leading to immune escape owing to T cell depletion (Dyck and Mills, 2017).

Knockdown/inhibition of LSD1 in cancer cells upregulates the expression of repetitive elements, including ERV, induces dsRNA stress and activates type 1 interferons, which stimulate anticancer T cell immunity and suppress cancer growth (Sheng et al., 2018). The expression of LSD1 in TNBC is inversely correlated with immunoregulatory factors such as cytotoxic T cell-attracting chemokine (C-C motif) ligand 5 (CCL5). Inhibition of LSD1 increases the expression of effector T cell-attracting chemokine factors and PD-L1 and promotes the trafficking of CD8^+^ T lymphocytes in the microenvironment of TNBC (Qin et al., 2019). Knockout of LSD1 or inhibition of LSD1 activity can also enhance the immune response of T cells in various cancers, such as cervical, ovarian, gastric, and oral cancers (Soldi et al., 2020; Xu et al., 2021; Alhousami et al., 2022; Shen et al., 2022). Therefore, LSD1 inhibition may be an effective adjuvant for immunotherapy in patients with poorly immunogenic cancers.

#### 3.1.9 Silent information regulator 1

Silent information regulator 1 (SIRT1) is a class III histone deacetylase that is dependent on nicotinamide adenine dinucleotide and is involved in the regulation of glucose and lipid metabolism, insulin secretion, oxidative stress, organ metabolism and tumorigenesis (Soni et al., 2021; Jalgaonkar et al., 2022). The highly conserved SWIRM domain of LSD1 interacts with SIRT to form a functional complex, which mediates the activity of SIRT1. The interaction between SIRT1 and LSD1 plays a conserved and cooperative role in H3K4 demethylation to inhibit Notch signalling-mediated gene transcription (Cao et al., 2021). SIRT1 and LSD1 are aberrantly expressed in cancers and play an antagonistic role in DNA repair and mutation acquisition (Boila and Sengupta, 2020; Wang et al., 2020). Moreover, the expression of LSD1 and SIRT1 is negatively correlated with the prognosis and survival of patients.

#### 3.1.10 Mammalian target of rapamycin complex 1

Mammalian target of rapamycin complex 1 (mTORC1) is an evolutionarily conserved serine/threonine protein kinase responsible for the regulation of cell proliferation and metabolism (Battaglioni et al., 2022; Huang et al., 2022). The activity of mTORC1 is regulated or feedback-regulated by a variety of intracellular signaling factors, such as growth factors, protein levels, energy levels, and intracellular oxygen partial pressure (Wang et al., 2021; Li et al., 2022). mTORC1 can participate in cancer progression by phosphorylating its substrates to regulate intracellular protein synthesis, material metabolism (lipid, nucleotide, and glucose) and autophagy.

mTORC1 is a key regulator of autophagy, and its activity is directly and positively correlated with the level of LSD1 (Li et al., 2019). Activated LSD1 alters the levels of phosphorylated mTOR1 and AKT through the mTOR signaling pathway, thereby reducing autophagy in tumour cells (Feng S. et al., 2016; Ma et al., 2022). Inhibition of LSD1 in drug-resistant leukaemia helps detect aberrantly activated mTORC1; therefore, activation of mTORC1 may be a pro-survival mechanism for cancer cells (Abdel-Aziz et al., 2020). Simultaneous knockdown or pharmacological inhibition of mTORC1 and LSD1 significantly enhanced primary cell differentiation and reduced the proportion of primary human AML cells (Deb et al., 2020).

#### 3.1.11 F-box and WD-40 domain protein 7

F-box and WD-40 domain protein 7 (FBXW7) is an important recognition factor in the ubiquitin–proteasome degradation pathway. It is also a typical cancer suppressor, which mediates the ubiquitination and degradation of various oncoproteins and regulates the occurrence and development of malignant cancers.

LSD1 recognises and binds to FBXW7 and inhibits its dimerisation, resulting in autoubiquitination/degradation of FBXW7 (Lan et al., 2019). Inhibiting the activity/expression of LSD1 exerts anticancer effects by stabilising/upregulating the accumulation of FBXW7. Knockdown/silencing of LSD1 or overexpression of FBXW7 in PCa cells can significantly reduce the activity of oncoproteins such as c-MYC and NOTCH-1. The oncogenic activity of LSD1 depends on the interaction of LSD1 with FBXW7, independent of its demethylase activity (Lan et al., 2019), which is supported by functional validation of the allosteric inhibitors SP-2509 and GSK-2879552 (Qin et al., 2020).

#### 3.1.12 Long noncoding RNAs

Long noncoding RNAs (lncRNAs) are non-protein-coding RNA molecules with a length of >200 nucleotides (Ghafouri-Fard and Taheri, 2020). They are involved in the growth, proliferation, metastasis and drug resistance of cancers and other related processes and are abnormally expressed in various cancers, such as gastric, lung, breast, liver, and endometrial cancers (Chen et al., 2016; Kim et al., 2018; Raeisi et al., 2019). LSD1 can bind to lncRNAs to exert oncogenic effects.

Enhancers of LSD1 interact with lncRNAs and are recruited to the promoter regions of cancer suppressors, such as KLF2, LATS2, and P21, to inhibit their transcriptional activity (Li W. et al., 2016; Zang et al., 2016). LSD1 can activate p62-mediated antioxidative pathways by upregulating certain lncRNAs (Ma et al., 2021). In addition, FOXP4-AS1 and DUXAP8 can recognise and bind to LSD1 and upregulate the expression of LSD1 to accelerate cancer progression (Chen et al., 2019; Gong et al., 2019).

### 3.2 Signaling pathways regulated by LSD1

#### 3.2.1 Energy metabolism pathway

The energy metabolism of cancer cells is significantly different from that of normal cells; that is, despite the presence of sufficient oxygen, cancer cells use glycolysis as the main energy production method, which is called the Warburg effect (Bose et al., 2021; Cook et al., 2021). Overexpressed LSD1 in cancer cells promotes glucose uptake and glycolytic activity and upregulates the expression of GLUT1 and glycolytic enzymes; however, it strongly downregulates the expression of mitochondrial metabolism-related genes (Luo et al., 2021). Knockdown or pharmacological inhibition of LSD1 can activate the transcriptional activity of the gluconeogenesis genes FBP1 and G6Pase, resulting in increased *de novo* glucose synthesis and decreased intracellular glycogen content (Wang D. et al., 2022). Inhibition of LSD1 activity in oesophageal cancer cells can significantly reduce the extracellular acidification rate (ECAR) and increase the oxygen consumption rate (OCR) and OCR/ECAR ratio (Kosumi et al., 2016). LSD1 may contribute to the malignant behaviour of oesophageal cancer by regulating metabolism, glycolytic pathway, and mitochondrial respiration. In addition, LSD1 plays an important role in regulating adaptive thermogenesis and lipid metabolism and may be a novel target for treating obesity (Wang D. et al., 2022).

#### 3.2.2 NOTCH signalling pathway

The highly conserved Notch signalling pathway regulates essential life processes such as cell differentiation, proliferation and angiogenesis *via* interaction among adjacent cells or paracrine/endocrine effects (Trindade and Duarte, 2020; Misiorek et al., 2021). Cancer cells use the Notch signalling pathway to form a microenvironment of cancer-promoting factors by promoting the secretion of cancer-related inflammatory cytokines, such as IFN-γ, which regulates immune cell function; and IL-1β and CCL2, which mediate immune infiltration, the release of senescence-related cytokines and immunosuppression, thereby suppressing the immune response (Cortesi et al., 2021; Wang L. et al., 2022; Zhou et al., 2022).

The interaction between LSD1 and NOTCH1 inhibits the expression and downstream signalling of NOTCH1. ORY-1001, a selective inhibitor of LSD1, can activate the Notch pathway and strongly inhibit cancer growth in chemotherapy-resistant PDX models (Augert et al., 2019). However, the expression of LSD1 and NOTCH3 in clinical specimens of HCC is strongly negatively correlated with the survival of patients. This negative correlation may result from the increased expression of LSD1 in cancer-associated fibroblasts (CAFs), which regulate driving NOTCH3-mediated self-renewal of cancer stem cells (Liu et al., 2018).

#### 3.2.3 PI3K/AKT signalling pathway

Phosphatidylinositol-3-kinase/protein kinase B (PI3K/Akt) is a well-known growth and development signalling pathway in organisms, which plays a crucial role in cell proliferation, apoptosis, cell cycle, DNA repair, and protein synthesis (Hoxhaj and Manning, 2020). PI3K/AKT not only mediates life activities such as the proliferation, migration, metastasis, and self-renewal of cancer cells but also regulates the self-renewal ability of cancer stem cells (Yang et al., 2020).

Silencing/knockout of LSD1 inhibited the invasion and metastasis of gastric cancer cells owing to the significant downregulation of VEGF-C, p-PI3K, PI3K, p-AKT, AKT, VEGFR-3, MMP-2, and MMP-9 (Pan et al., 2019). LSD1 can enhance AR signalling and activate the PI3K/AKT pathway in PCa by increasing p85 gene expression in the absence of AR (Wang et al., 2019). Similarly, TCP, an inhibitor of LSD1, can block the binding of LSD1 to the promoter regions of NOTCH3, Hes1, and CR2, thereby reducing Notch and PI3K/Akt/mTOR signalling (Hou et al., 2019). Moreover, high expression of LSD1 was detected in patients with colorectal cancer with mutations in the catalytic subunit of PI3K, and PIK3CA-mutated colorectal cancer cells were found to be dependent on LSD1 for growth (Miller et al., 2020).

#### 3.2.4 Wnt/β-Catenin signalling pathway

The Wnt/β-Catenin signalling pathway plays a key role in regulating cell pluripotency and carcinogenesis and is closely related to important processes such as cancer proliferation, metastasis and chemotherapy resistance (Zhang and Wang, 2020; Liu et al., 2022). LSD1 can activate the Wnt/β-Catenin signalling pathway by downregulating DKK1 in colorectal cancer. After pharmacological inhibition or silencing of LSD1, the translocation of β-Catenin to the nucleus is significantly reduced, and the transcription of the target gene c-Myc is downregulated. Combination therapy with LSD1 inhibitors and 5-FU strongly inhibited Wnt/β-Catenin signalling and DNA synthesis, significantly inhibiting the proliferation, migration, and growth of cancer cells in a tumor xenograft-bearing model (Peng et al., 2020). LSD1 is necessary to emerge cancer stem cells after long-term sorafenib treatment. LSD1 inhibitors downregulate the expression of multiple regulators of the Wnt/β-Catenin signalling pathway and increase the sensitivity of cancer cells to sorafenib (Huang et al., 2017). LSD1 can downregulate the Wnt pathway antagonists APC2 and DKK1 through demethylation in thyroid cancer, thus inhibiting APC2 transcription and activating the HIF-1α/DKK1 axis to regulate cancer progression (Zhang W. et al., 2022).

## 4 Conclusion and outlook

As the first discovered histone demethylase, LSD1 has promoted the research progress of epigenetics. LSD1 is a key regulator of the proliferation, apoptosis, differentiation, invasion, metastasis, drug resistance, and cancer stemness and hence is a promising therapeutic target for cancers (Feng Z. et al., 2016; Wang et al., 2016; Alsaqer et al., 2017; Cusan et al., 2018; Verigos et al., 2019; Tayari et al., 2021).

This review summarises the cancer-promoting regulatory mechanisms of LSD1 in lung, breast, gastric, colorectal, liver, bladder, leukaemia, and other cancers. LSD1 regulates different signalling pathways, such as the Wnt/β-Catenin, PI3K/AKT signalling, EMT-related, Notch signalling and ubiquitin–proteasome pathway, and target proteins, such as the classic cancer suppressor star protein p53, HIFs, and immune checkpoints, in different types of cancer to mediate cancer progression. However, because the occurrence and development of cancers are very complex biological processes, all oncogenic factors or pathways mediated by LSD1 could not be described in this review. Some examples include SOX2, which maintains cancer cell stemness; sex hormone receptors (AR and ER); the transcriptional repressor ZNF516; retinoic acid-related orphan receptor alpha (RORα); aryl hydrocarbon receptors; ubiquitin-specific protease 28 and miRNAs (Park et al., 2016; Bai et al., 2017; Khanal et al., 2017; Kim et al., 2017; Li et al., 2017; Macheleidt et al., 2018; Cuyàs et al., 2020; Yu et al., 2020; Zhang et al., 2021). However, the detailed mechanisms of LSD1 underlying carcinogenesis remain unclear and warrant further investigation. For example, does or how LSD1 is involved in ferroptosis and pyroptosis, and whether LSD1 interact with SITR6?

To date, many LSD1 inhibitors have been reported, with some having excellent anticancer activity. Among these inhibitors, ORY-1001 and GSK2879552 have entered clinical trials, thus validating the clinical application of LSD1 as a therapeutic target for cancers and incredibly encouraging the development of strategies targeting LSD1 for treating cancers. Furthermore, inhibiting LSD1 protein synthesis or promoting its degradation may be a potential anticancer strategy (Qi et al., 2020). Tumorigenesis patterns may be induced by point-to-net or multi-point-to-net cascades involving numerous regulatory factors/signalling pathways. In the future, a cure for cancer may be developed if LSD1-like master switches involved in the regulation of cascade reactions related to tumorigenesis and cancer development or target proteins with relatively more regulatory functions can be identified or if a multitarget regulation strategy can be developed. Therefore, refining the oncogenic mechanism of LSD1 will help develop promising tumor therapeutic drugs or strategies.
